# Supplementary material for: Long-term outcomes of hospital survivors following an ICU stay: A multi-centre retrospective cohort study
Source: PLoS One. 2022 Mar 28;17(3):e0266038. doi: 10.1371/journal.pone.0266038 (PMC8959167; doi:10.1371/journal.pone.0266038)
Supplement: S1 Fig — * This criterion requires a variable only available for matched patients. Its calculation was performed prior to the researchers receiving receipt of the data. Therefore, if an admission for an individual was one of the excluded unmatched admissions it would still have been considered when this variable was created. ANZICS; Australian and New Zealand Intensive Care Society, VAED; Victorian Admitted Episode Dataset. (DOCX) [file pone.0266038.s001.docx]

**Supporting Information:**

| **S1 Fig. Inclusion and exclusion process.** |
| --- |
| 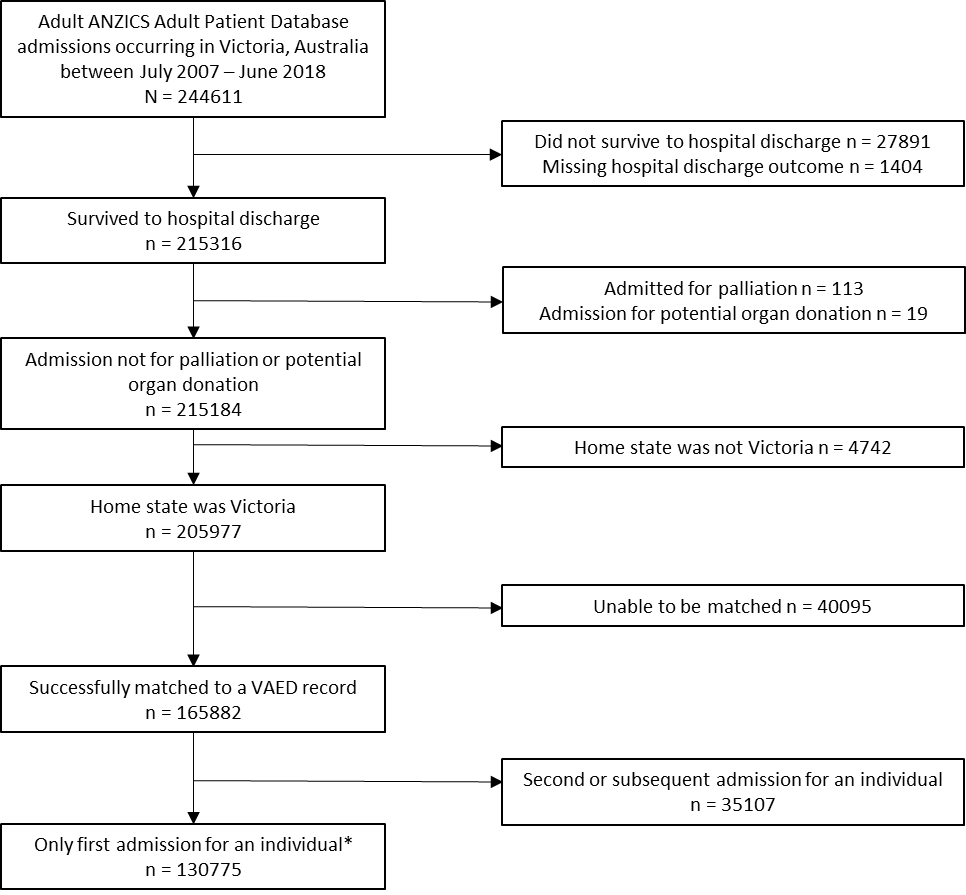 |
| * This criterion requires a variable only available for matched patients. Its calculation was performed prior to the researchers receiving receipt of the data. Therefore, if an admission for an individual was one of the excluded unmatched admissions it would still have been considered when this variable was created. ANZICS; Australian and New Zealand Intensive Care Society, VAED; Victorian Admitted Episode Dataset. |
