# Supplementary material for: Long-term outcomes of hospital survivors following an ICU stay: A multi-centre retrospective cohort study
Source: PLoS One. 2022 Mar 28;17(3):e0266038. doi: 10.1371/journal.pone.0266038 (PMC8959167; doi:10.1371/journal.pone.0266038)
Supplement: S2 Fig — Follow-up is only 3-years post hospital discharge to allow comparison between each year period. Three-year period groups were created to facilitate simple comparisons. (DOCX) [file pone.0266038.s002.docx]

| **S2 Fig. Survival curves limited to 3-years post discharge stratified by year group (solid lines) compared to the matched standard Australian population (dotted lines).** |
| --- |
| 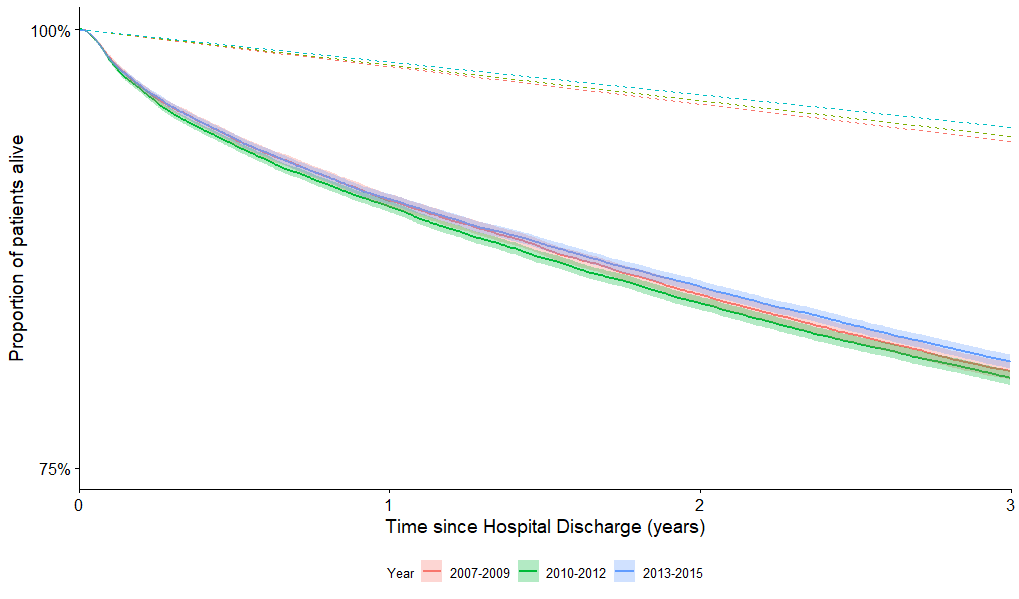   \| **Year period** \| **Study Cohort (95% CI) 3-year survival** \| **Difference from baseline** \| **Matched Cohort 3-year survival** \| **Difference from baseline** \| \| --- \| --- \| --- \| --- \| --- \| \| 2007 – 2009 \| 80.5% (80.0% - 81.0%) \| Baseline \| 97.9% \| Baseline \| \| 2010 - 2012 \| 80.1% (79.7% - 80.5% \| -0.4% \| 98.0% \| +0.1% \| \| 2013 - 2015 \| 81.0% (80.6% - 81.4%) \| +0.5% \| 98.1% \| +0.2% \| |
| Follow-up is only 3-years post hospital discharge to allow comparison between each year period. Three-year period groups were created to facilitate simple comparisons. |
