# Supplementary material for: Long-term outcomes of hospital survivors following an ICU stay: A multi-centre retrospective cohort study
Source: PLoS One. 2022 Mar 28;17(3):e0266038. doi: 10.1371/journal.pone.0266038 (PMC8959167; doi:10.1371/journal.pone.0266038)
Supplement: S3 Fig — The 95% confidence intervals of the study cohort lines are represented by the shaded areas. Groups: Readmissions (N = 130707), Admission type (N = 129830). (DOCX) [file pone.0266038.s003.docx]

| **S3 Fig. Survival curves of stratified by readmission status or admission type (solid lines) compared to the matched standard Australian population (dotted lines).** |
| --- |
| 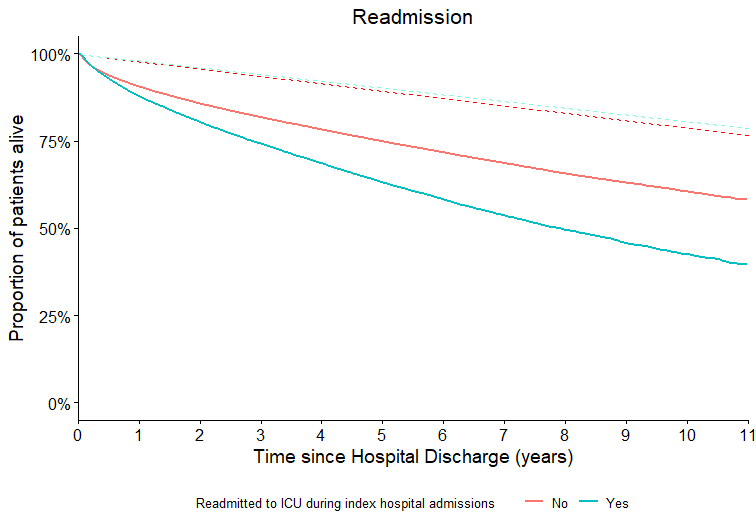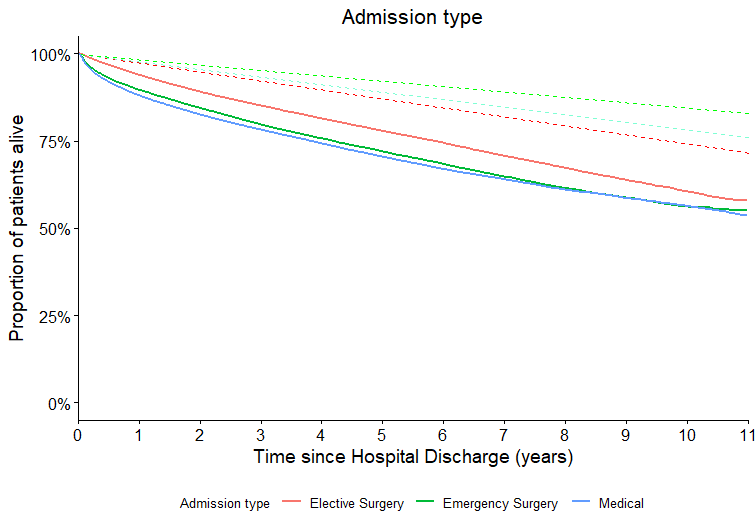 |
| The 95% confidence intervals of the study cohort lines are represented by the shaded areas.  Groups: Readmissions (N=130707), Admission type (N=129830). |
