# Supplementary material for: Long-term outcomes of hospital survivors following an ICU stay: A multi-centre retrospective cohort study
Source: PLoS One. 2022 Mar 28;17(3):e0266038. doi: 10.1371/journal.pone.0266038 (PMC8959167; doi:10.1371/journal.pone.0266038)
Supplement: S1 Table — The unlinked cohort had the same inclusion criteria applied to it as the linked cohort. Variables that appeared in the Table 1 of the main manuscript that are absent from this table were those not included in both datasets. IQR; Interquartile range, Index of Relative Socioeconomic Advantage and Disadvantage, ICU; Intensive Care Unit, ED; Emergency Department, LOS; Length of stay, APACHE; Acute Physiological and Chronic Health Evaluation, COPD; Chronic Obstructive Pulmonary Disease, GI; Gastrointestinal. (DOCX) [file pone.0266038.s004.docx]

| **S1 Table:** **Comparison of characteristics between patients matched to the VAED and those not able to be matched.** |
| --- |
| \| **Characteristic** \| **Matched, N = 130775** \| **Not Matched, N = 40095** \| **p-value** \| \| --- \| --- \| --- \| --- \| \| **Age (years)** \| 64.0 (49.0, 74.8) \| 64.8 (49.4, 75.7) \| <0.001 \| \| Sex \|  \|  \|  \| \| Male \| 77089 (58.9%) \| 22797 (57.0%) \|  \| \| Female \| 53686 (41.0%) \| 17108 (42.8%) \|  \| \| Unknown \| 0 \| 64 (0.1%) \|  \| \| **IRSAD score (median, IQR)** \| 983 (937, 1,037) \| 979 (937, 1,024) \| <0.001 \| \| Unknown \| 903 (0.6%) \| 874 (2.1%) \|  \| \| **Patient area of residence** \|  \|  \| <0.001 \| \| Major City \| 74083 (56.6%) \| 19457 (48.5%) \|  \| \| Inner Regional \| 39790 (30.4%) \| 15108 (37.7%) \|  \| \| Outer Regional \| 11246 (8.5%) \| 4567 (11.4%) \|  \| \| Remote \| 139 (0.1%) \| 60 (0.1%) \|  \| \| Unknown \| 5517 (4.2%) \| 903 (2.3%) \|  \| \| **Admission post Medical Emergency Team call** \| 12691 (9.7%) \| 5662 (14.1%) \| <0.001 \| \| Unknown \| 5349 (4.0%) \| 1582 (3.9%) \|  \| \| **Limitations of medical therapy at ICU admission** \|  \|  \| <0.001 \| \| No limitations \| 121279 (92.7%) \| 36490 (91.0%) \|  \| \| Limitations \| 4697 (3.5%) \| 2264 (5.6%) \|  \| \| Unknown \| 4799 (3.6%) \| 1341 (3.3%) \|  \| \| **Year of admission** \|  \|  \| <0.001 \| \| 2007-2009 \| 25125 (19.2%) \| 7031 (17.5%) \|  \| \| 2010-2012 \| 35793 (27.3%) \| 8732 (21.8%) \|  \| \| 2013-2015 \| 38835 (29.6%) \| 12323 (30.7%) \|  \| \| 2016-2018 \| 31022 (23.7%) \| 12009 (30.0%) \|  \| \| **Hospital type** \|  \|  \| <0.001 \| \| Tertiary \| 68878 (52.6%) \| 8687 (21.7%) \|  \| \| Metropolitan \| 37101 (28.3%) \| 15263 (38.1%) \|  \| \| Rural / Regional \| 24796 (18.9%) \| 16145 (40.3%) \|  \| \| **Admission type** \|  \|  \| <0.001 \| \| Emergency \| 84566 (64.6%) \| 28702 (71.6%) \|  \| \| Elective \| 45332 (34.6%) \| 11157 (27.8%) \|  \| \| Unknown \| 877 (0.6%) \| 236 (0.6%) \|  \| \| **Admitted to ICU from** \|  \|  \| <0.001 \| \| Theatre \| 62785 (48.0%) \| 15621 (39.0%) \|  \| \| ED \| 41086 (31.4%) \| 1789 (4.5%) \|  \| \| Ward \| 16776 (12.8%) \| 15263 (38.1%) \|  \| \| Hospital Transfer \| 10128 (7.7%) \| 7296 (18.2%) \|  \| \| Unknown \| 0 (<0.1%) \| 21 (0.3%) \|  \| \| **Length of ICU stay (days)** \| 1.8 (0.9, 3.5) \| 1.8 (0.9, 3.3) \| <0.001 \| \| Unknown \| 20 (<0.1%) \| 22 (<0.1%) \|  \| \| **Length of hospital stay (days)** \| 8.3 (4.7, 15.1) \| 9.5 (4.8, 19.1) \| <0.001 \| \| Unknown \| 2 (<0.1%) \| 3648 (9.1%) \|  \| \| **Invasively ventilated during admission** \| 55303 (42.2%) \| 12154 (30.3%) \| <0.001 \| \| Unknown \| 3945 (3.0% \| 2151 (5.4%) \|  \| \| **APACHE III-J score (median, IQR)** \| 49 (36, 64) \| 49 (35, 65) \| <0.001 \| \| Unknown \| 624 (0.4%) \| 317 (0.8%) \|  \| \| **Discharged home** \| 91700 (70.1%) \| 27722 (69.1%) \| <0.001 \| \| **Diagnosis on admission to ICU** \|  \|  \| <0.001 \| \| Cardiac (Surgical) \| 19997 (15.2%) \| 4048 (10.1%) \|  \| \| Cardiac (non-Surgical) \| 8980 (6.8%) \| 3333 (8.3%) \|  \| \| Cardiac arrest \| 2419 (1.8%) \| 552 (1.4%) \|  \| \| Vascular/Thoracic surgery \| 7966 (6.0%) \| 1858 (4.6%) \|  \| \| Abdominal aortic aneurysm \| 2324 (1.7%) \| 374 (0.9%) \|  \| \| Respiratory \| 6477 (4.9%) \| 2343 (5.8%) \|  \| \| COPD \| 2669 (2.0%) \| 1005 (2.5%) \|  \| \| Pneumonia \| 5703 (4.3%) \| 1984 (4.9%) \|  \| \| Sepsis (excluding Pneumonia) \| 9438 (7.2%) \| 3933 (9.8%) \|  \| \| Neurological \| 3354 (2.5%) \| 1044 (2.6%) \|  \| \| Stroke / Intra-cerebral Haemorrhage \| 1673 (1.2%) \| 630 (1.6%) \|  \| \| Sub-arachnoid Haemorrhage \| 1090 (0.8%) \| 231 (0.6%) \|  \| \| Seizure \| 1998 (1.5%) \| 789 (1.9%) \|  \| \| Orthopaedic (non-spinal) \| 4084 (3.1%) \| 1371 (3.4%) \|  \| \| Orthopaedic (spinal) \| 631 (0.4%) \| 82 (0.2%) \|  \| \| Trauma (head) \| 3076 (2.3%) \| 590 (1.5%) \|  \| \| Trauma (non-head) \| 5953 (4.5%) \| 1485 (3.7%) \|  \| \| Toxicological (overdose) \| 6275 (4.7%) \| 2341 (5.8%) \|  \| \| GI Surgery \| 16976 (12.9%) \| 5081 (12.7%) \|  \| \| GI Medical \| 4331 (3.3%) \| 1794 (4.5%) \|  \| \| Surgical (other) \| 7976 (6.0%) \| 2226 (5.6%) \|  \| \| Medical (other) \| 7385 (5.6%) \| 3001 (7.5%) \|  \| |
| The unlinked cohort had the same inclusion criteria applied to it as the linked cohort.  Variables that appeared in the Table 1 of the main manuscript that are absent from this table were those not included in both datasets.  IQR; Interquartile range, Index of Relative Socioeconomic Advantage and Disadvantage, ICU; Intensive Care Unit, ED; Emergency Department, LOS; Length of stay, APACHE; Acute Physiological and Chronic Health Evaluation, COPD; Chronic Obstructive Pulmonary Disease, GI; Gastrointestinal. |
