# Supplementary material for: Long-term outcomes of hospital survivors following an ICU stay: A multi-centre retrospective cohort study
Source: PLoS One. 2022 Mar 28;17(3):e0266038. doi: 10.1371/journal.pone.0266038 (PMC8959167; doi:10.1371/journal.pone.0266038)
Supplement: Attachment — Submitted filename: Reviewer comments.docx [file pone.0266038.s005.docx]

Reviewer #1

| **Comment** | **Response** |
| --- | --- |
| 1. *The length of stay in ICU seems to be short, can you add the data of SOFA score as organ damage at the time of ICU admission?* | We thank the reviewer for their comment.  In this paper we have used the APACHE-IIIJ score as a marker of illness severity. The study database collects variables specifically to calculate this score. We have calculated a SOFA score and added it to Table 1 to provide further information regarding the cohorts severity of illness.  Of note the median ICU length of stay in the study (1.8 days) was like that reported for all ICU admissions in Australia throughout the 2019/2020 period. (1.7 days) (<https://www.anzics.com.au/wp-content/uploads/2021/09/2020-ANZICS-CORE-Report.pdf>)  This length of stay was the median for our full cohort. It must be considered that 34.6% of admissions were elective, which in many cases are short admissions for close monitoring in the immediate post-operative period. |
| 2. *Do the authors have any data about the length of ventilation? Please add the data in Table 1 and 2.* | We thank the reviewer for their comment.  We have added a row to *Table 1* to report the median length of ventilation for ventilated patients.  We have not added this variable to *Table 2* as it is only applicable for a subset of the included cohort. We believe an analysis of ventilated patients specifically is a valuable area of future research.  Of note we did not add it to *Appendix Table S1* as it is a variable only available for patients that were linked to the VAED. |
| 3. *Most of the patients discharged from ICU have lower quality of life due to ICU-acquired weakness and are often transferred to rehabilitation facilities or chronic care hospitals. What do you think is the reason why the discharge rate to home is so high in this study?* | We thank the reviewer for their comment.  Our study reported that 70.1% of patients were discharged home. The other 29.9% of patients survived to discharge but were discharged to other locations. (Rehabilitation etc.) Due to a change in the way the discharge location variable was coded during our study period we were unable to present this data.  Our rate of discharge home is similar to other large studies of ICU patients in first-world countries. A study of 155 ICUs in the US had a slightly lower rate of 63.3%. [1] Another US study of over 280 ICUs had a discharge home rate of 57-65% (no overall rate was given – only rates for specific time periods). [2] In this study patients that did not survive to discharge were included, ~13% of patients died in this study, meaning the 57-65% figure is an underestimation of the true discharge home rate.  [1] <https://www.ncbi.nlm.nih.gov/pmc/articles/PMC4212937/>)  [2] https://www.ncbi.nlm.nih.gov/pmc/articles/PMC4057290/ |
| 4. *I recommend that the following three suggestions to make the Table 1 and 2 easier to read: to delete the ruled line surrounding the title of Table 1 and 2, to delete the vertical line in the table, and to delete all horizontal lines of the table but the first, second, and bottom.* | We thank the reviewer for their comment.  We have implemented the suggested changes. |

Reviewer #2

| **Comment** | **Response** |
| --- | --- |
| 1. *Considering that all ICU stays could be included, the ratio of elective admissions seems normal but I am surprised by the short reported median ICU length of stay and the quite low use rates of mechanical ventilation (MV) and renal replacement (CRRT). A comment about these points could be added.* | We thank the reviewer for their comment.  We have added the following to our limitations section:  “*Finally, it must be considered by all readers that our cohort may be different from the patients they care for, and the whole group outcomes may differ significantly to the outcomes for specific patient cohorts.* *This is particularly relevant for countries with different healthcare systems to Australia. Of note our rates of mechanical ventilation and renal replacement therapy were low. The fact our study only included patients that survived to hospital discharge is likely a contributor to this.”* |
| 2. Although much less used than MV, CRRT seems also related to a protective effect like MV. The authors could more comment on these seemingly paradoxical results. | We thank the reviewer for their comment.  We have included mention of CRRT in the below text in our discussion section:  *“Similarly, the association of invasive ventilation and renal replacement therapy with good long-term survival was also unexpected and may be an example of survivorship bias as our study only included patients that left hospital alive.”* |
| 3. The authors performed analysis of multiple admission diagnosis sub groups but an analysis of the outcome of overall medical and overall surgical patients should be also interesting. In the same way, comparison of the outcome of patients with multiple ICU stays vs single stay should be performed. | We thank the reviewer for their comment.  We have included an analysis of medical and surgical (elective and emergency separated) patients in the appendix as a survival curve.  We have done the same for patients with multiple ICU stays. We stratified the cohort into those that were readmitted to ICU during the same admission and those that weren’t. When referring to multiple ICU stays, we have defined this as a readmission during the same admission (rather than in another hospital admission) to prevent the introduction of immortal time bias. |
| 4. The study address only survival and not quality of life or disability. This point is stated by the authors but could be more highlighted. | We thank the reviewer for their comment.  We have added to our existing discussion of this issue in our limitations:  “Sixthly, our study only reported survival. Previous work has found high rates of self-reported disability and low rates of returning to work at six-months post ICU admission. (30, 31) Therefore, it is important to consider that survival does not necessarily equate with an acceptable quality of life for all patients.”  We also highlight our mention of this issue with respect to elderly cardiac surgery patient as an area for research in our future directions section:  “Additionally, investigation into whether the superior survival outcomes of this group extend to quality of life is warranted. “ |
| 5. As stated by the authors, results of the study should be considered when discussing an ICU admission; however these results are derived from a large sample (which is a strength), with differences between sub groups related to some factors. So, it seems reasonable to be cautious in the face of excessive generalisation, particularly in case of hyperspecialized center or very particular type of patients. | We thank the reviewer for their comment.  In our response to comment #1 we have discussed this issue:  “*Finally, it must be considered by all readers that our cohort may be different from the patients they care for, and the whole group outcomes may differ significantly to the outcomes for specific patient cohort. This is particularly relevant for countries with different healthcare systems to Australia. Of note our rates of mechanical ventilation and renal replacement therapy were low. The fact our study only included patients that survived to hospital discharge is likely a contributor to this.”* |

Reviewer #3

| **Comment** | **Response** |
| --- | --- |
| 1. *The way I understand the study is that only patients who left the hospital were evaluated for the long-term outcome. However, there were certainly patients who left the ICU but died in hospital. I could not find this information, which I believe, is of importance. Hence, the title of the manuscript is not really adequate; a title such as “Long-term outcomes of patients who left the hospital after an ICU stay” looks more appropriate.* | We thank the reviewer for their comment.  We have altered the title of our work to: “*Long-term outcomes of hospital survivors following an ICU stay: a multi-centre retrospective cohort study”*  The inclusion of only patients that survived to hospital discharge was described in the study population section of the methods |
| 2. *The authors report on just under half of patients were invasively ventilated (line 240-241). Does this include elective post-operative patients? If so, they should be removed as this MV does not really match those on MV bc of respiratoy failure or other critical condition. This might affect what is written in the discussion section (line 359-361) and the result in table 2.* | We thank the reviewer for their comment.  The variable examining invasive ventilation status includes patients that were ventilated for any reason, this included elective post-operative patients.  Unfortunately, the database used for this study does not record the reason for invasive ventilation. Therefore, it is not possible to determine which elective post-operative patients remained ventilated in ICU for the purposes of routine post-operative care or due to unexpected respiratory compromise.  In the future it would certainly be valuable to make this distinction, however, at this time it is not possible with our data. The primary aim of our study was to describe the long-term outcomes of all survivors following an ICU admission (both elective and emergency).  To add further context to the characteristics of the cohort we have reported the proportion of elective surgical admissions that were invasively ventilated in Table 1. |
| 3. *The authors compare mortality between “ICU survivors” and an aged-matched population. Not really surprising as those admitted in ICU probably suffer from more comorbidities. This is perfectly demonstrated with the COPD burden.* | We thank the reviewer for their comment.  We agree that those in ICU are likely to have far more comorbidities than the matched population by virtue of being in hospital. The inclusion of a matched cohort was to provide a baseline of expected survival that readers could compare to. We believe this makes the finding that elderly cardiac surgery patients have better/equivalent survival to the matched cohort particularly noteworthy and is grounds to examine this area specifically in the future. |
| 4. The reason to take cardiac surgical patients as the reference for the Multivariate Cox regression for long-term mortality hazard remains unclear. | We thank the reviewer for their comment.  The cardiac surgery patients were included as the reference group as they were the largest diagnosis group. This makes the analysis more stable than if a smaller group were used. This was done for all other groups except in the case of their being another strong reason to make a smaller group the reference category (ie: year of admission – choosing the earliest year group as the reference category).  We have added the following into the statistical methods to reflect this:  “The largest group for each variable was chosen as the reference group except in the case of year of admission where the earliest year group was chosen.” |
| 5. Likewise, some of the following results are quite difficult to understand: for instance, why would head trauma patients display lower long term mortality thatn cardiac surgical patients? Are they younger? | We thank the reviewer for their comment.  We agree there are several results that were unexpected. The multivariable cox regression controlled for all included variables. This means the difference between cardiac surgery and head trauma is independent of any age differences.  We intend to examine these sub-groups specifically in the future to determine the underlying factors contributing to the difference. |

Reviewer #4

| **Comment** | **Response** |
| --- | --- |
| 1. *This cohort represents 53,5% of ICU admissions of the period! This should be clarified in the manuscript. One point is, that 6,25% didn´t survive the hospital stay and 16,8% were admitted for a second ICU stay it has to be pointed out that 21% of the patients could not be matched. Therefore, the strengths of this very good and important study is limited as authors demonstrate in table S1 that in all parts values are significantly different between matched and not matched cases. Therefore generalisability should be* | We thank the reviewer for their comment.  We have reviewed the method in which we reported the proportion of patients matched between the databases. To report a more accurate rate we applied all inclusion criteria (except the removal of readmissions) to our original dataset. Readmissions were not removed as the variable for the criterion was only available for matched admissions. We elected to follow this method as previously patients that were not eligible for inclusion (ie: they did not survive to discharge) were included in our calculation of a match rate. We have also updated our inclusion figure to reflect this method.  After applying the criteria, we had 205,977 patients eligible for inclusion. Of these 165,344 were matched (match rate = 80.5%). Finally, readmissions were removed resulting in our final study cohort of 130,775 patients.  It should be noted the variable to identify readmissions reports an “admission number” and was derived prior to the researchers receiving the dataset. Therefore, application of the inclusion criteria does not impact the identification of which admissions were readmissions.  We have added the following text to our methods to explain this process:  *“The match rate was calculated by dividing the admissions successfully matched after all inclusion criteria were applied (except readmissions) by all admissions after the same inclusion criteria were applied. (S1 Fig) The readmission criteria were not applied as the variable for this was only available to matched admissions.”*  We have then reported the following in our results:  *“Using the previously described method, 80.3% of eligible ICU admissions in the ANZICS APD were able to be matched to a VAED record.”*  To reiterate to readers the limitation brought about by 19.7% of patients not being matched we highlight the following section in the limitations:  “Firstly, the linkage between the ANZICS APD and the VAED was only 80.3%. However, there were few clinically significant differences between the linked and unlinked groups, and both groups had identical median APACHE III-J scores.”  Whilst all variables did have significant differences between the matched and unmatched cohort, it should be noted these differences were very small. Of particular note the median APACHE-IIJ score and median age were very similar between groups. Due to the large sample size even small differences were significant in our analysis. |
| 2. *Line 359 “Similarly, the association of invasive ventilation and good long-term survival was also unexpected and may be an example of survivorship bias as our study only included patients that left hospital alive.” For this statement it would be useful to look on the data, how many of these ventilated patients came from OR after elective surgery, as 48% of patients where admitted from OR. These patients have a priori a better chance of survival as documented by APACHE or SAPS score demonstrate.* | We thank the reviewer for their comment.  Of those invasively ventilated 38.8% were elective admissions.  Of those not invasively ventilated 28.4% were elective admissions.  The association between invasively ventilation status and survival controlled for admission type and severity of illness on top of all other variables. Therefore, the association was independent of these two factors.  We believe that specifically examining the patients who were invasively ventilated is a potential area for future research. |
| 3. *Line 365 “This study provides clinicians with objective information to inform discussions with patients and family members about the implications of admission to ICU and long-term survival after critical illness.” This statement has to been seen with concern. The study data provide a hint, if patients survive hospital admission, what may be the future. There several limitations of the data matched for this analysis (e.g. see point one). Therefore, it may be useful to look on the patients who not survived ICU stay, according to the underlying diseases. Further Australia has its own way, whether patients will be admitted to ICU, or not. Meaning, there are many patients admitted to ICU in other regions of the world who would never be admitted to ICU in Australia.* | We thank the reviewer for their comment.  Our study only aimed to examine the long-term survival of patients that survived to hospital discharge. The ANZICS APD has been used previously in several papers to examine the short-term survival of patients across a variety of cohorts. We believe by focussing on long-term survivors we have been able to shed light on a group not yet studied in detail, particularly in the Australian context. Unfortunately, examining the non-survivors to hospital discharge is beyond the scope of our manuscript.  With respect to the international differences in thresholds for admission to ICU we agree with the reviewer. To reiterate this point to readers we have included the following to our limitations section:  *“Finally, it must be considered by all readers that our cohort may be different from the patients they care for, and the whole group outcomes may differ significantly to the outcomes for specific patient cohorts. This is particularly relevant for countries with different healthcare systems to Australia.”* |
| 4. *Line 382 “Secondly, our follow-up period was 11.1 years, with a median of 3.6 years.” This should be put into respect, that this is only correct for patients admitted in 2007 and not for the hole study population.* | We thank the reviewer for their comment.  We have amended the text in the results section to read:  “Follow-up ranged from 0 days (the patient died immediately following discharge) to 11.1 years post-discharge (for those admitted in 2007), with a median of 3.6 years post-discharge.” |
